# Association between Chronic Disease Self-Management, Health Status, and Quality of Life in Older Taiwanese Adults with Chronic Illnesses

**DOI:** 10.3390/healthcare10040609

**Published:** 2022-03-24

**Authors:** Tung-Chen Han, Huey-Shyan Lin, Ching-Min Chen

**Affiliations:** 1 Department of Nursing, College of Medicine, National Cheng Kung University, Tainan 701401, Taiwan; x00003210@meiho.edu.tw; 2 Department of Nursing, Meiho University, Neipu 912009, Taiwan; 3 Department of Health-Business Administration, Fooyin University, Kaohsiung 83102, Taiwan; sc035@fy.edu.tw

**Keywords:** self-management, health status, quality of life, frailty, mediator

## Abstract

Aging is accompanied by many chronic comorbidities and disabilities, and entails medical expenses, which affects the quality of life among older adults. The purpose of this study was to investigate whether the health status of older adults with chronic diseases mediates chronic disease self-management to predict quality of life. Methods: This research adopted a cross-sectional correlation study design. Convenient sampling was performed in outpatient departments commonly visited by older adults in a medical center in Southern Taiwan. The following measures were collected: (1) Physiological measurement: left handgrip, right handgrip, and lower extremities’ muscle strength. (2) Questionnaires: cognitive function was measured by the Alzheimer’s disease (AD)-8 scale, possible frailty with the Kihon Checklist (KCL), functional status with the Barthel Index (BI) and the Lawton and Brody Instrumental Activities of Daily Living (IADL) scales, and self-management for chronic disease and quality of life with the (WHOQOL)-BREF, Taiwan version. Results: Chronic disease self-management is correlated with health status and is directly related to quality of life. Chronic disease self-management also indirectly affects quality of life through health status (cognitive status and risk of frailty), showing that health status partly mediates the correlation between chronic disease self-management and quality of life. Conclusions: A health status feedback system should be introduced in related chronic disease self-management measures for older adults so that they can be aware of their own health status and so that their quality of life is improved. Custom-made nursing interventions are necessary for the reduction in or delay of disability or risk of frailty in older adults, thereby enhancing their quality of life.

## 1. Introduction

Aging leads to decreased physical activity, mood disorders, social isolation, and increased risk of chronic diseases. With increases in age, frailty accompanied by cognitive disorders may occur in older adults, and the deterioration of organ functions may increase the negative effects on the body, causing reductions in independent living skills [1] and loss of autonomy [2], in turn affecting the quality of life of older adults [2]. In addition, older adults with chronic diseases may also experience worse cognitive functions, which become an obstacle when making treatment decisions. This also leads to poor medical compliance, preventing older adults from participating in daily physical activities [3,4], and increasing physiological and emotional pain as well as the complexity of care, which further influence the quality of life of older adults [1,3,5]. A study on physical activities, frailty, and health-related quality of life of the elderly in the community in Taiwan found that, for the elderly, higher scores of physical activity were related to a higher quality of life, while higher scores of frailty were related to a lower quality of life, and the quality of life was significantly affected by physical activity and frailty status [1]. Some studies have also indicated that cognitive executive functions and wellbeing were significantly correlated [6]. The result that patients with higher cognitive functions had a higher quality of life was also found in another study of patients with rheumatic diseases [3].

Prolonging life is a very important public health goal that emphasizes quality of life and independent living [7]. Many countries have used quality of life as a major health indicator [8]. It is very important to confirm the quality of life of an individual, and priority should be given to public health decision making and clinical research activities. When older adults are able to maintain mobility and overall independence in terms of activities, a sense of security and wellbeing is generated, which could be reflected in a broader quality of life instead of merely a health-related quality of life [6]. Healthcare providers should set older adults’ quality of life as their primary goal of care [9]. Aging and chronic diseases affect health-related quality of life, so it is necessary to pay attention to the treatments and interventions being carried out among older adults [1] to help them manage chronic diseases and undergo interventions that lead to the development of their self-management abilities, enabling them to be able to maintain or improve their quality of life [9]. It is necessary to not only treat diseases but also to learn to engage in disease management [3]. Only by continuous management could symptoms be reduced, functionality be maintained, and the prevention of complications be possible, that is, disease management is even more important than disease treatment [8].

Self-management is crucial for improving the health and quality of life for older adults with chronic diseases [10]. Looking at self-management from the perspective of aging typically means examining declines in reserves and resources in various areas. These losses typically reinforce each other, where a small loss in one area can lead to a spiral of loss of resources in multiple areas [11]. Overall self-management has been defined as the generative capacity of managing an individual’s crucial resources [12]. Loss-related self-management is crucial in the aging process and is an effective strategy for not only responding to losses but also for engaging actively in managing important resources that support and maintain a sense of wellbeing [13]. Looking at self-management from the perspective of diseases, the concept of self-management is indispensable for maintaining health and managing diseases, where, ultimately, self-management interventions all enhance the ability to improve one’s health status [14]. Self-management refers to activities of engaging in health, building physical reserves, and preventing adverse after-effects; interactions with healthcare providers and compliance with recommended treatment plans; monitoring physical and emotional status as well as making appropriate management decisions based on the results of self-monitoring; and the management of the impact of disease on a patient’s self-esteem, the ability to play an important role, and the relationships with others [15], thereby improving their quality of life [16].

Many studies have pointed out that self-management may have impacts on physical functions, cognitive functions, and quality of life. For example, a cross-sectional study explored the correlation among the self-management ability, frailty, and perceived poor health among older adults in the community. The results showed a significant correlation among self-management ability, frailty, and perceived poor health. Self-management ability was negatively correlated with perceived poor health, while frailty was positively correlated with perceived poor health [17]. One early study explored the relationships among social, cognitive, and physical functions, self-management ability, and wellbeing among hospitalized older adults, and also evaluated the mediation effect of self-management on wellbeing. It was found in the results that functions (social, cognitive, and physical functions) are related to wellbeing, and that self-management ability was related to social, cognitive, and physical functions, in addition to wellbeing, indicating that self-management had mediation effects on social, cognitive, and physical functions, in addition to wellbeing. Older adults with lower social, cognitive, and physical functions had poorer self-management ability than those with higher functioning [18]. Another study explored the ability to predict the degree to which self-management affects the physical health of older adults with cardiovascular disease, diabetes mellitus, or obstructive pulmonary disease, and it was found that the level of self-management was a predictor of physical health, and that older adults with higher levels of self-management had better physical health outcomes [19]. Another study explored the relationships among health behavior, degree of self-management, physical health, depressive symptoms, and the well-being of older Turkish immigrants, and the results showed that a physically active status was positively correlated with overall wellbeing, and that self-management ability was positively correlated with physical health and wellbeing [20]. The development of self-management skills has been proven to successfully help individuals manage their diseases and improve their health outcomes [14].

Previous literature shows that higher self-management is correlated to a better health status and higher quality of life, and that a higher health status is associated with a better quality of life. However, studies on how older adults achieve a satisfactory quality of life or wellbeing, and whether it is achieved through other mediators, are not common. Thus, the purpose of this study was to examine the relationship between self-management and quality of life, and investigate the mediating effect of health status among older adults with chronic illness in Taiwan. Understanding the mediation of health status to self-management and quality of life would benefit the development of new interventions in the future.

## 2. Methods

### 2.1. Design

A cross-sectional correlation study design was used.

### 2.2. Setting and Sample

Convenience sampling was adopted, and participants were recruited from among the older adults in the neurology, cardiology, metabolism, rehabilitation, and family medicine outpatient departments in hospitals in Southern Taiwan (26). Subjects were eligible for the study if they met the following criteria: (1) older adults with a confirmed diagnosis of chronic diseases, (2) those aged 60 years and older, (3) those with no diagnosed dementia, disability, hearing problems, or communication disabilities, (4) those who were able to communicate in Chinese or Taiwanese, and (5) those who agreed to participate in the study. The sample size was estimated using SAMPLE POWER version 2.0 software (SPSS Inc., Chicago, IL, USA), in which the statistical power and signiﬁcance were set at 0.80 and 0.05, respectively. The study adopted the medium level of R^2^ (0.13) of Cohen’s (1988) gold standard to estimate multiple correlations. The required sample size was calculated to be 98 participants. Because we anticipated receiving invalid questionnaires, an additional 10% of the sample (10 participants) was added, for an estimated sample size of 108 participants.

### 2.3. Data Collection

Study samples were selected by the research team through their prearranged physician appointments. The RAs reviewed clients’ charts first, and then held discussions with physicians concerning candidate participants who met the inclusion criteria. Then, cases were invited by an RA to participate while waiting for their appointment. Subjects were interviewed in person, and the data were collected in outpatient departments. Surveys and physiological measurements were conducted by trained research assistants. The research assistants were given instructions and interviewer training for consistency before the implementation of this research project to ensure the accuracy of the data.

### 2.4. Measures

#### 2.4.1. Demographics

Baseline attributes included sex, age, marital status, living conditions, religious beliefs, level of education, work, and economic conditions. Disease characteristics included disease diagnoses, number of diseases, and Charlson comorbidity index (CCI) scores. The CCI was developed by Charlson and colleagues in 1987 as a comorbid severity adjustment tool to control for comorbid diseases before treatment or surgery and to track patient prognosis. It has been well-validated, and can be used to calculate the total score for 19 disease conditions for predicting one-year mortality: the scores range from 0 (no condition) to 6. A higher score indicates a more severe comorbidity burden [21].

#### 2.4.2. Health Status

Functional status. In this study, the Barthel Index (BI) and the Lawton and Brody Instrumental Activities of Daily Living (IADL) scales are utilized. The BI scale measures performance in ten activities of daily living (ADLs). Each respondent’s response is divided into the “with help” and “independent” categories. The total score ranges from 0 to 100, where a higher score indicates higher functional performance in ADLs [22]. The BI scale exhibits high reliability, with a Cronbach’s α of 0.89 [23]. The Cronbach’s α in this study was 0.88. In the present study, instrumental activities of daily living (IADLs) [24] were used to reflect the level of functional performance [24]. This scale comprises a total of 8 items. The IADL score for each item ranges from 2 to 4, with a total score of 24, where a higher score indicates a higher ability to perform IADLs. This scale exhibits high reliability, with a Cronbach’s α of 0.92 [23]. The Cronbach’s α in this study was 0.86.

Physiological measurement. In this study, vision, hand grip, and lower extremity muscle strength were measured. The chair stand test was used as a measure of muscle strength. The participants were asked to stand up from a chair from a sitting position and then to sit down again, and the number of whole sets completed within 30 s was calculated [25] to measure lower extremity muscle strength. A dynamometer made in Japan was used for measuring the upper extremity grip strength. The participants were asked to take the grip strength test with the dynamometer three times each for both hands, and the average measured value was used for the data analysis [26].

Possible frailty. The Kihon Checklist (KCL), developed by the Japanese Ministry of Health, Labor, and Welfare, was used to identify older adults at risk of requiring care/support. The KCL comprises 25 items (yes/no) divided into seven domains: activities of daily living (items 1–5), physical strength (items 6–10), nutrition (items 11–12), oral function (items 13–15), socialization (items 16–17), memory (items 18–20), and mood (items 21–25), and has been used for screening frailty among older adults [27]. The sum of all indices ranges from 0 (no frailty) to 25 (severe frailty). A participant is identified as showing frailty if they obtain a score of 10 points or more in the lifestyle section of the KCL. A score of 3 or more indicates low physical strength in the respective domain, and a score of 2 indicates a low nutritional status in the respective domain. A score of 2 or more in the oral function domain suggests low oral functioning. A negative answer to question number 16 indicates “house-bound”; a score of 1 or more in the memory domain suggests low cognitive function; and, finally, a score of 2 or more in the mood domain indicates a risk of depression [28], where a higher score indicates worse functioning [29]. The results of the KCL can be analyzed separately based on each domain [28], where a higher score in each domain of the checklist indicates a higher risk of requiring support or care in that domain [27,28,29]. The KCL exhibits satisfactory internal consistency (Cronbach’s α = 0.78) [29]; the Cronbach’s α in the present study was 0.80.

Cognitive function. Cognitive status was assessed using the Alzheimer’s disease (AD)-8 scale. This scale is mainly used to detect early symptoms of dementia in adults [30]. A total of eight items are used to detect changes in the respondents’ memory, problem-solving abilities, orientation, and daily activity performance. The number of “Yes” responses to the questions are totaled. A total score ranging from 0 to 1 indicates normal cognitive function, and a score of 2 or more indicates impaired cognition, thereby indicating a need for further diagnosis. The Cronbach’s α for the reliability of this scale is 0.87 [30]. The Cronbach’s α obtained in this study was 0.82.

#### 2.4.3. Chronic Disease Self-Management

The chronic disease self-management scale was developed based on the related literature [31,32]. It consists of four domains: partnership (eight items), performance of self-care (22 items), problem solving (four items), and handling emotions (five items). The respondents were asked to fill out the questionnaire based on their own self-management of diseases in the previous three months. The scores range from 0 (never managed in the manner specified) to 1 (sometimes), 2 (often), and 3 (always). A higher score indicates more efficient disease self-management. This scale had satisfactory internal consistency in this study (Cronbach’s α = 0.82). The chronic disease self-management scale was developed according to the reviewed literature. The scale has been evaluated by five experts with expert validity. The S-CVI/UA, universal agreement in this study was 0.89. The item-level CVI (I-CVI) ranged from 0.6 to 1.0, which demonstrated a good validity.

#### 2.4.4. Quality of Life (QoL)

In this study, the World Health Organization’s (WHO) Quality of Life (WHOQOL)-BREF, Taiwan version, [33], was used. This scale comprises a total of 28 questions, including 24 items on subjective feelings about life in the physical, psychological, social relationship, and environment domains, two items measuring overall quality of life and general health, and two local questions, with a score ranging from 4–20 points for each domain, where a higher score indicates a higher quality of life. The Cronbach’s α for the reliability of the scale reached 0.91, while the Cronbach’s α for each domain ranged between 0.70 and 0.77 [33]. The Cronbach’s α for the reliability of the scale in the present study reached 0.91, while that for each domain ranged between 0.71 and 0.79.

### 2.5. Ethical Approval

After this study was approved by the institutional review board (IRB; approval number B-ER-100-388), the study subjects were given a study consent form, after which the researcher monitored the subjects’ progress during the study period. The researcher explained the purpose of the research and process to each research subject, who were allowed to withdraw at any time. The interviewees’ rights and interests were assured. The data were applied only in this study, and were not made public.

### 2.6. Data Analysis

Data were analyzed using SPSS-V22 software. Descriptive statistics were used to summarize demographic data. The relationships between variables were tested using the Pearson correlation coefficient (г). For further analysis, a path analysis was applied to explore the mediating effects of the health status on the antecedent (chronic disease self-management) and the outcome (quality of life) variables. In this study, α = 0.05 was set as the criterion for significance in the statistical analysis.

## 3. Results

### 3.1. Participants’ Characteristics

A total of 115 participants were included in this study, of which three were excluded because of incomplete data. The majority of the participants were women (62.3%), with an average age of 72.6 ± 7.49 (range of 60 to 99) years. Most of the participants were married (78.9%), and 91 participants (83.3%) lived with their families. In terms of religious beliefs, 45.9% were Buddhists, and most participants (53.7%) had a high school education or higher. More than 90% of the participants did not have a full-time job. The average total number of diseases among the participants was 1.6, with 58.9% having at least one disease and 27.7% having two diseases. The most prevalent chronic disease was hypertension, followed by cardiovascular disease, diabetes, hyperlipidemia, arthritis, fractures, and cataracts. In terms of the severity of disease, 50 participants (44.6%) scored “0”, followed by 24 (21.4%) scoring four, as demonstrated using an age-adjusted CCI (Table 1).

### 3.2. Health Status

The average score for ADLs was 98.7 ± 6.1 (range of 50 to 100), and that for IADLs was 22.0 ± 3.4 (range of 4 to 24). Only 42.9% of participants did not present early symptoms of dementia, as measured using the AD-8. The results revealed that 10 (8.9%) participants exhibited a risk of frailty in the lifestyle domain, whereas 24 (21.4%), 1 (0.9%), 26 (23.2%), 16 (14.3%), 58 (51.8%), and 30 (26.8%) of the participants had poor physical strength, a poor nutritional status, low oral functions, were house-bound and isolated, had low cognitive functions, and a risk of depression, respectively (Table 2). However, in terms of overall general frailty based on the total score, 39 participants (34.8%) exhibited frailty or an elevated risk of needing long-term care. In terms of handgrip strength, 68.2% and 51.4% of the participants had abnormal right and left handgrip strength, respectively. Up to 25.6% of the participants had abnormal lower extremity muscle strength, with an average of 16.9 ± 7.92 (range of four to 40) sit-downs and stand-ups within 30 s.

### 3.3. Chronic Disease Self-Management and Quality of Life

Table 3 presents the scores for chronic disease self-management. The scoring index for chronic disease self-management among the study participants was 56.49, indicating moderate chronic disease self-management. A score of 72.27 was obtained in the overall quality of life domain, indicating that the participants had a high quality of life.

### 3.4. Relationships among Chronic Disease Self-Management, Health Status, and Quality of Life

In this study, a Pearson correlation was first applied to explore the correlation between chronic disease self-management, health status, and quality of life. Regarding health status, left and right handgrip strengths, cognitive function, risk of frailty, IADLs, and ADLs were individually significantly correlated with disease self-management (r = 0.26, 0.27, −0.33, −0.35, 0.33, and 0.21, respectively, *p* = 0.001–0.05). In addition, left and right handgrip strengths, cognitive function, risk of frailty, IADLs, and ADLs and quality of life were significantly correlated (r = 0.26, 0.26, −0.50, −0.56, 0.37, 0.34, and 0.44, *p* = 0.001) (Table 4).

### 3.5. Mediating Effects of the Health Status on the Relationship between Chronic Disease Self-Management and Quality of Life

**Figure 1:**
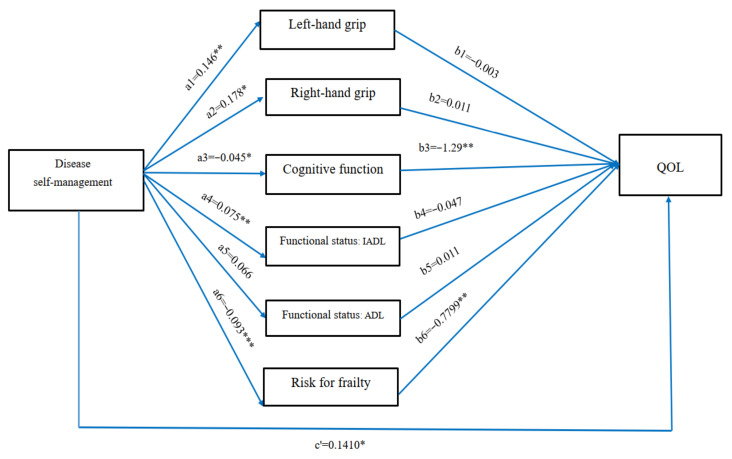
Model of the mediating roles played by maintaining health among older adults with chronic illness. Model showing the mediating role of maintaining health status and the relationship between disease self-management and quality of life. a = Direct effect of independent variable (disease self-management) on mediator (maintaining health status). b = Direct effect of mediator (maintaining health status) on outcome variable (quality of life). c’ = Direct effect of independent variable (disease self-management) on outcome variable (quality of life). * *p* < 0.05, ** *p* < 0.01, *** *p* < 0.001.

This study was conducted to examine the mediating effect of health status on the relationship between the antecedent variable (chronic disease self-management) and the outcome variable (quality of life). After controlling for personal attributes and disease characteristics, the path analysis showed that chronic disease self-management had a significant impact on items related to the health status of the subjects (coefficients: a1 = 0.146, *p* = 0.008, a2 = 0.178, *p* = 0.003, a3 = −0.045, *p* = 0.007, a4 = 0.075, *p* = 0.000, and a6 = −0.0933, *p* = 0.000), with the exception of ADLs (a5 = 0.066, *p* = 0.669). Among the health status items, only cognitive function (*b*3 = −1.29, *p* = 0.003) and risk of frailty (*b*6 = −0.77, *p* = 0.005) had a significant impact on quality of life. Pathway a and pathway *b* indicated that efficient chronic disease self-management could generate a favorable health status (except for ADLs), and that a lower risk of impaired cognition function and frailty could lead to a high quality of life. The bootstrap method used by Preacher and Hayes [34] was utilized to examine the indirect effects of the independent variables on the dependent variables, based on the significance of the proposed mediators. If the 95% confidence intervals (CIs) of the ab coefficient did not include zero, this indicated that the indirect effect was significant. The multiple pathways for which the 95% CI of the ab coefficient did not include zero were cognitive function (95% CI = 0.0237–0.0975) and risk of frailty (95% CI = 0.0130–0.1518), demonstrating that cognitive function and risk of frailty were mediators of chronic disease self-management and quality of life. Because chronic disease self-management exerted significant effects on overall quality of life (c = 0.27, *p* ≤ 001), the correlation between chronic disease self-management and quality of life achieved significance (c’ = 0.1410, *p* = 0.018) after the health status was controlled for. Thus, the mediating effects of cognitive function and risk of frailty accounted for 21.5% and 26.0%, respectively, of the total effect (a3 × b3/c = 21.5%, a6 × b6/c = 26.0%) (Figure 1). The personal attribute and disease characteristic variables, as well as chronic disease self-management and health status, accounted for 44.9% (R^2^ = 0.449) of the variance in quality of life (Figure 1).

## 4. Discussion

### 4.1. Mediating Effect of Health Status on the Relationship between Chronic Illness Self-Management and Quality of Life

To the best of our knowledge, this is the first study on the correlation among older adults’ level of self-management, health maintenance, and quality of life. It provides new insights by considering the complex relationships among self-management, health maintenance, and quality of life. The study results showed that chronic disease self-management can both directly and indirectly affect the quality of life of older adults with chronic diseases, and that cognitive function and risk of frailty can partly mediate the correlation between self-management and quality of life, indicating that those with efficient self-management have fewer cognitive risks and frailty problems in addition to a higher quality of life. Recent studies have indicated that cognitive impairment has a direct impact on self-management [3,18], such as monitoring symptoms, adhering to medication plans, and the development of as well as adherence to a lifestyle intended to maintain one’s state of health [35]. It was shown in this study that those with higher self-management scores had better cognitive abilities, mainly because when performing self-management it was necessary to improve attention, memory, and problem-solving strategies, as well as the visuospatial skills required for self-monitoring (such as blood glucose tests) [36]. In addition, constant practice and repetition were required to enhance skills such as memory, attention, and executive functions [37], and various cognitive compensatory strategies were also used to embed new strategies into habits and routines [36]. In addition, the subjects had to monitor their self-condition every day by keeping a diary or through the use of other methods, which made it possible for them to objectively review their behavior in terms of meeting their goals, adjusting their behavior, and strengthening their self-management strategies [38]. Three levels of prevention were employed to integrate health behavior and the self-management of chronic diseases into daily life that could thereby improve cognitive ability [39]. This study did not directly examine and test the domains of specific objective cognitive functions. It is recommended that future studies incorporate the impact of self-management on cognitive functions. It was shown in this study that cognitive functions had significant direct impacts on quality of life, similar to the results found in other studies [3,18,40]. This was because cognitive executive functions decline with age, and complete executive ability is an important ability to complete health promotion behaviors such as medication management, diet and lifestyle changes, self-monitoring responses, and undergoing professional follow-ups. It was more likely for those with high cognitive executive functions to accomplish such goals [8] and improve their near- and long-term quality of life [41]. It could be inferred that the AD-8 questionnaire in this study involved both memory and concentration, where the subjects could experience memory problems and inattention that could, in turn, lead to doubts as to their self-control ability and further generate an adverse effect on their quality of life [40]. The findings of this study indicated that physical functions had a significant direct impact on quality of life, similar to the findings of other studies [42,43]. Physical health is one of the important measures of quality of life, and improvements in physical performance, where the development of independence may change the perspective of older adults on their disease status, which in turn reduces the impact of their disease, improving quality of life [42]. This study showed that self-management and quality of life are significantly correlated, similar to the results found in other studies [43]. Self-management ability can be modified, and it is related to improvements in quality of life through self-management interventions, such as health education, lifestyle education, improvements in the level of knowledge of chronic diseases, and the promotion of exercise.

### 4.2. Relationships between Health Status, Chronic Disease Self-Management, and Quality of Life

The results of the present study indicate that the adequacy of one’s living allowance is correlated with quality of life, indicating that those with adequate living allowances had a higher quality of life than those with inadequate living allowances, which is consistent with the findings of other researchers [44]. Retirement causes the income of older adults to decrease, while health-related costs tend to increase [12]. Extended life expectancy indicates that financial resources should be properly managed over an extended life span [45]. Life after retirement could last for decades, and a fixed income limits the ability of an individual to cope with sudden changes in economic wellbeing [46], thus affecting quality of life. Comorbidity was found to be correlated with quality of life, consistent with the results of other studies [44], indicating that comorbidity not only limits physiological functions but also affects other aspects of quality of life. The results revealed a significant positive correlation between upper extremity muscle strength (grip test) and quality of life, consistent with the results of previous studies [47,48]. The measurement of muscle grip is an indicator of overall muscle tone, and, since aging contributes to a wide range of body system changes, it is a suitable marker in the aging process. In addition, hand grip is a useful single marker for general weakness and bio-aging [48]. The decline in function caused by the loss of muscle strength leads to a decline in older adults’ maximum function, thereby making it difficult for them to perform daily tasks [49]. The results of the present study indicated a significant positive correlation between daily activities and quality of life, which is consistent with the results of other studies [50,51]. A decline in individuals’ physiological functions, caused by various factors, results in the loss of social support and social contact, thereby reducing quality of life [51]. The findings of the present study showed a significant negative correlation between the risk of frailty and quality of life, which is consistent with the conclusions of another study [52]. Major functional and psychosocial disability limits the performance of ADLs and social roles among older adults, resulting in a poorer self-perceived quality of life [52]. The present study results revealed that early dementia had a significant negative correlation with quality of life, which is consistent with the results of another study [53]. Cognitive impairment is a barrier to performing self-care and may be an important contributor to increasing mortality and a reduction in quality of life [54]. Functional and cognitive decline have been shown to be associated not only with a loss of independence and reduced quality of life but also with an increased use of health services, a greater risk of institutionalization, and a greater risk of mortality [55]. The present study revealed a significant positive correlation between chronic disease self-management and quality of life. The participants applied self-management skills in their daily life to assess symptoms and make lifestyle changes in terms of the management of medication, diet, and exercise, as well as reductions in the use of tobacco and alcohol. This could empower older adults to incorporate their preferences to control their own lives to achieve a higher quality of life and restore their maximum functions [56,57].

## 5. Suggestions and Limitations

To prevent the consequences of chronic diseases as well as the limitations and obstacles caused by old age, and to improve quality of life, multiple interventions are required. Before and after performing a self-management intervention in older adults whose frailty and cognitive functions may be affected by such interventions, the important thing is to include the screening of their frailty status in addition to their physical and cognitive functions related to routine self-care, as well as to follow-up changes in frailty status and physical and cognitive functions [4]. Due to the complexity of cognitive processes, attention, memory, reasoning, judgement, executive function, and problem-solving abilities must be incorporated into interventions, particularly when an individual is cognitively impaired, where the adoption and maintenance of self-management skills will be affected by cognitive impairment and may have adverse impacts on quality of life [3]. Therefore, multidimensional self-management skills were added in the training of self-management skills, and education concerning lifestyle, regulatory skills, and proactive coping was also covered [12,19], which could have the unexpected effect of delaying cognitive and functional impairment as well as improving quality of life [1,5,6]. The results of this study suggest that related health status indicators should be monitored to understand the impact of controlling disease on health outcomes and quality of life. Furthermore, the concept of self-management for older adults with chronic diseases can be integrated into nursing education to equip nursing students with adequate knowledge with which to guide older adults with chronic diseases to engage in self-management in the future.

Interpretation of the results of this study is limited by several factors. First, the cross-sectional nature of the study limits generalization. The sample of this cross-sectional study came from outpatients, and could be biased. It could be an overestimation of the patients’ self-management. We would suggest that a future study could be initiated to investigate a sample apart from outpatient clients. In addition, health status and chronic disease self-management are affected by multiple factors. Therefore, future research involving longitudinal studies are needed. Second, only 15 (13.4%) of the participants were considered to have multimorbidity. These data are relatively low compared to that of Taiwan. The low proportion could be due to small samples. Future studies should include more subjects so as to reduce biases. Third, the participants in this study were not limited to a specific chronic disease, and therefore the range of diseases was wide. Subjects with different diseases may have different self-management strategies, health statuses (cognitive and physical functions), and qualities of life, which is also a limitation of this study. Finally, this study used the AD-8scale to subjectively measure the cognitive status of the participants instead of using a neuropsychological test battery to capture the specific category of cognitive impairment. In the future, the participants’ cognitive status should be objectively measured, and subjects in need of help in various dimensions of quality of life, such as physiology, psychology, society, and environment, should be identified.

## 6. Conclusions

To the best of our knowledge, this study is the first to clarify that health status plays a mediating role in the correlation between chronic disease self-management and quality of life among older adults with chronic diseases. Chronic disease self-management was negatively correlated with very early dementia and risk of frailty, which were the health status variables. Negative correlations were noted between health status (i.e., cognitive function and risk of frailty) and quality of life. A positive correlation was noted between chronic disease self-management and quality of life. The next steps will be to evaluate these factors in interventions to confirm the findings of these results. 

## Figures and Tables

**Table 1:** Demographic characteristics of participants (*n* = 112).

Variable	Number of Subjects	(%)
Gender		
Male	42	37.5
Female	70	62.3
Age Mean: 72.58 ± 7.49 Median: 72 Range: 60 to 99		
60–65 years old	18	16.1
66–70 years old	32	28.6
71–75 years old	21	18.8
76–80 years old	25	22.3
Eighty-one years old and over	16	14.3
Marital status		
Single/separated/divorced/widowed/other	23	20.5
Married	89	78.9
Living status (whether living with others)		
No	18	16.1
Yes	94	83.9
People living together (number of people) Mean: 2.63 ± 1.93 Median: 2 Range: 0 to 10		
Living alone	18	16.1
Two people	56	50.0
Three people or more	38	33.9
Religious belief		
None	20	17.9
Buddhism	53	47.3
Taoism/folk beliefs	23	20.5
Christianity/Catholicism	16	14.3
Level of education		
Literate/illiterate	9	8.0
Primary school	25	22.3
Middle school/junior high school	20	17.9
Senior high school/vocational school	22	19.6
Junior college	10	8.9
College (inclusive) and above	26	23.2
Employed		
No	104	92.9
Yes	8	7.1
Main source of income		
Children/spouse/parents/others	46	41.1
Pension	56	50.0
Government grants	10	8.9
Adequacy of living allowance		
Abundant and in surplus	30	26.8
Generally enough	71	63.4
Not enough	11	9.8
Number of diseases Mean: 1.60 ± 0.94 Median: 1 Range: 1 to 6		
One	66	58.9
Two	31	27.7
Three and more	15	13.4
CCI (after age adjustment) Mean: 2.39 ± 2.37 Median: 3 Range: 0 to 9		
0	50	44.6
2	2	1.8
3	14	12.5
4	24	21.4
5	14	12.5
Six and more	8	7.2

**Table 2:** Cognitive function and risk of frailty in the older adults with chronic disease (*n* = 112).

Scale/Subscale (Number of Items)	Risk as Showing Score	Number (%)	Mean (SD)
AD8 (8)			1.59 ± 2.11
0		48 (42.9)	
1		24 (21.4)	
≥2	(Early symptoms of dementia)	40 (35.7)	
Kihon Checklist (25)			
Physical strength (5)	≥3 (low physical strength)	24 (21.4)	1.38 (±1.39)
Nutrition (2)	2 (low nutritional status)	1 (0.9)	0.26 (±0.46)
Oral function (3)	≥2 (low oral function)	26 (23.2)	1.0 (±0.92)
Socialization (1)	Negative answer on No. 16 (house-bound)	16 (14.3)	0.14 (±0.35)
Memory (3)	≥1 (low cognitive function)	58 (51.8)	0.75 (±0.84)
Mood (5)	≥2 (depression risk)	30 (26.8)	1.03 (±1.38)
Lifestyle (20)	≥10 (frailty)	10 (8.9)	4.42 (±3.48)
Total KCL score (25)	≥7 (general frailty or elevated risk of needing LTCI service)	39 (34.8)	5.46 (±4.32)

**Table 3:** Chronic disease self-management and quality of life in the older adults (*n* = 112).

Scale (Possible Range of Scores)	Mean	SD	Minimum	Maximum	Score Index
Disease self-management ^a^ (0 to 117)	65.99	16.78	23	95	56.49
Partnership (0 to 24)	18.31	5.75	5	24	75.56
Performance of self-care activities (0 to 66)	31.40	8.57	6	54	47.58
Problem solving (0 to 12)	8.2	3.29	1	12	68.38
Emotion handling (0 to 15)	8.9	3.34	2	15	74.53
Overall QoL ^b^	57.82	8.32	23.56	78.43	72.27
Physical domain (4 to 20)	14.59	2.47	7.43	20	72.72
Psychological domain (4 to 20)	14.19	2.49	6	20	70.95
Social relationships domain (4 to 20)	14.28	2.29	9	20	71.44
Environment domain (4 to 20)	14.92	2.17	7.56	20	74.62

^a^ Score index of chronic disease self-management = mean/117 × 100. Score index of partnership = mean/24 × 100; score index of performance of self-care activities = mean/66 × 100; score index of problem solving = mean/12 × 100; and score index of emotion handling = mean/15 ×100. ^b^ Score index of quality of life = mean/80 × 100.

**Table 4:** Correlations among chronic disease self-management, health status, and quality of life.

Variable	(1)	(2)	(3)	(4)	(5)	(6)	(7)	(8)	(9)	(10)	(11)
(1) Living expenses	-										
(2) Comorbidity	0.05	-									
(3) ADL	−0.15	−0.10	-								
(4) IADL	−0.08	0.07	0.71 **	-							
(5) Risk for frailty	0.28 **	0.26 **	−0.57 **	−0.65 **	-						
(6) Very early dementia	0.20 **	0.23 **	−0.34 **	−0.38 **	0.49 **	-					
(7) Left handgrip	−0.19 *	0.03	0.16	0.25 **	−0.40 **	−0.25 **	-				
(8) Right handgrip	−0.11	0.06	0.17	0.32 **	−0.40 **	−0.24 *	0.87 **	-			
(9) Lower extremities muscle strength	−0.02	−0.19	0.15	0.29 **	−0.21	−0.24 *	0.25 *	0.26 *	-		
(10) Chronic self-management	−0.00	−0.08	0.21 *	0.33 **	−0.35 **	−0.33 **	0.26 **	0.27 **	0.10	-	
(11) Quality of life	−0.32 **	−0.20 *	0.34 **	0.37 **	−0.56 **	−0.50 **	0.26 **	0.26 **	0.44 **	0.18	-

* *p* < 0.05; ** *p* < 0.01. (1) (2) Spearman correlation.

## Data Availability

Not applicable.
